# Effect of Face Masks on Interpersonal Communication During the COVID-19 Pandemic

**DOI:** 10.3389/fpubh.2020.582191

**Published:** 2020-12-09

**Authors:** Nour Mheidly, Mohamad Y. Fares, Hussein Zalzale, Jawad Fares

**Affiliations:** ^1^Department of Communication and Journalism, Autonomous University of Barcelona, Barcelona, Spain; ^2^Faculty of Medicine, American University of Beirut, Beirut, Lebanon; ^3^College of Medical, Veterinary and Life Sciences, University of Glasgow, Glasgow, United Kingdom; ^4^Faculty of Medical Sciences, Neuroscience Research Center, Lebanese University, Beirut, Lebanon; ^5^Department of Neurological Surgery, Feinberg School of Medicine, Northwestern University, Chicago, IL, United States

**Keywords:** SARS-CoV-2, coronavirus, communication, social distancing, pandemic (COVID-19), pandemic

## Abstract

Interpersonal communication has been severely affected during the COVID-19 pandemic. Protective measures, such as social distancing and face masks, are essential to mitigate efforts against the virus, but pose challenges on daily face-to-face communication. Face masks, particularly, muffle sounds and cover facial expressions that ease comprehension during live communication. Here, we explore the role of facial expressions in communication and we highlight how the face mask can hinder interpersonal connection. In addition, we offer coping strategies and skills that can ease communication with face masks as we navigate the current and any future pandemic.

## Introduction

The COVID-19 pandemic has severely affected the way people communicate with each other. Precautionary measures to limit the spread of the virus necessitated a shift in the communication paradigm when it comes to greetings and handshakes. The arising situation required people to adopt salutations that do not entail physical contact, such as the “peace sign,” the “hand on chest,” and the “namaste” (1). In addition, emphasis on personal spaces and social distancing markedly increased, with telecommunication witnessing a huge rise, as business meetings, conferences, and educational activities shifted to virtual communication via social applications, such as Zoom, Cisco Webex, Skype, and Microsoft Teams.

Face-to-face communication, specifically, was majorly affected by the pandemic. The need for face masks, as an important protective measure to decrease the spread of the virus, had a huge toll on interpersonal communication. Facial expressions and gestures play a major role in facilitating interpersonal communication, comprehension, and the delivery of intended messages. As such, wearing face masks hindered the ability of seeing and understanding people's expressions during conversations, and decreased the impact of communicated material.

In this piece, we explore the role of facial expressions in communication and we highlight how the face mask can affect it. In addition, we offer coping strategies to enhance the quality of interpersonal communication while wearing protective face masks.

## Role of Facial Expressions in Communication

Facial expressions play a prominent role in communication and relay of emotion across individuals. People perceive facial expressions off one another, and this helps them forecast events and situations, and develop responses to them (2). The face, as an anatomical figure, can be separated into upper, middle, and lower portions, with each playing an important role in expressing the feelings and moods of an individual (3). For example, actions like smiling and grimacing involve lower facial structures, like the mouth, the lips, and the cheeks, and these are often included in our daily conversations.

Facial expressions of different emotions involve action units, or elementary changes in facial appearance recognized by the Facial Action Coding System, which is a system that taxonomizes human facial movements by their appearance on the face. These facial expressions are produced by a set of facial muscles (4). The middle face involves the “nose wrinkle,” an action unit that wrinkles and pulls the skin upward along the sides of the nose; this is used to convey disgust (4, 5). The lower face involves multiple action units, and these include the “chin raiser,” the “lip stretcher,” the “lip tightener,” the “lips part,” and the “jaw drop,” and each is associated with a set of facial muscles that convey a specific emotion (4, 5). The “chin raiser” pushes the boss of the chin and the lower lip upward, while the “lip tightener” causes lips to appear narrower; both action units are used to convey anger (4, 5). The “lips stretcher” stretches lips horizontally, and the “lips part” separates them to a limited extent; both action units are used to convey fear (4, 5). In addition, the “jaw drop” parts lips so that the space between the teeth is visible and this is used to convey surprise (4, 5).

The middle and lower face are noted to be very influential with regards to emotional recognition. Kestenbaum explored the modes of processing of emotional expression in children and showed that the mouth can be used to recognize a neutral expression and is best for recognizing the emotion of happiness (6). Gagnon et al. investigated children's ability to recognize fear, surprise, disgust, and anger based on information from the upper, middle, or lower face, and found that children can recognize fear, surprise, and anger using expressions involving the lower face, and disgust using expressions involving the middle face (5). While the upper face is also pivotal for the development of emotional expressions, the roles of the middle and lower face cannot be understated.

## Masking Facial Communication

The high infectivity of SARS-CoV-2 and the increasing rates of COVID-19 infection pushed physicians and health experts to recommend wearing facemasks during the pandemic. This measure combined with social distancing and handwashing helps in slowing the spread of the virus and decreasing its transmission, especially between people that are designated as asymptomatic carriers (7, 8). Previous studies comparing non-fit-tested P2 masks, surgical masks, and no masks in fighting influenza for households had shown that masks may reduce the transmission of viruses during pandemics (9).

**Figure 1:**
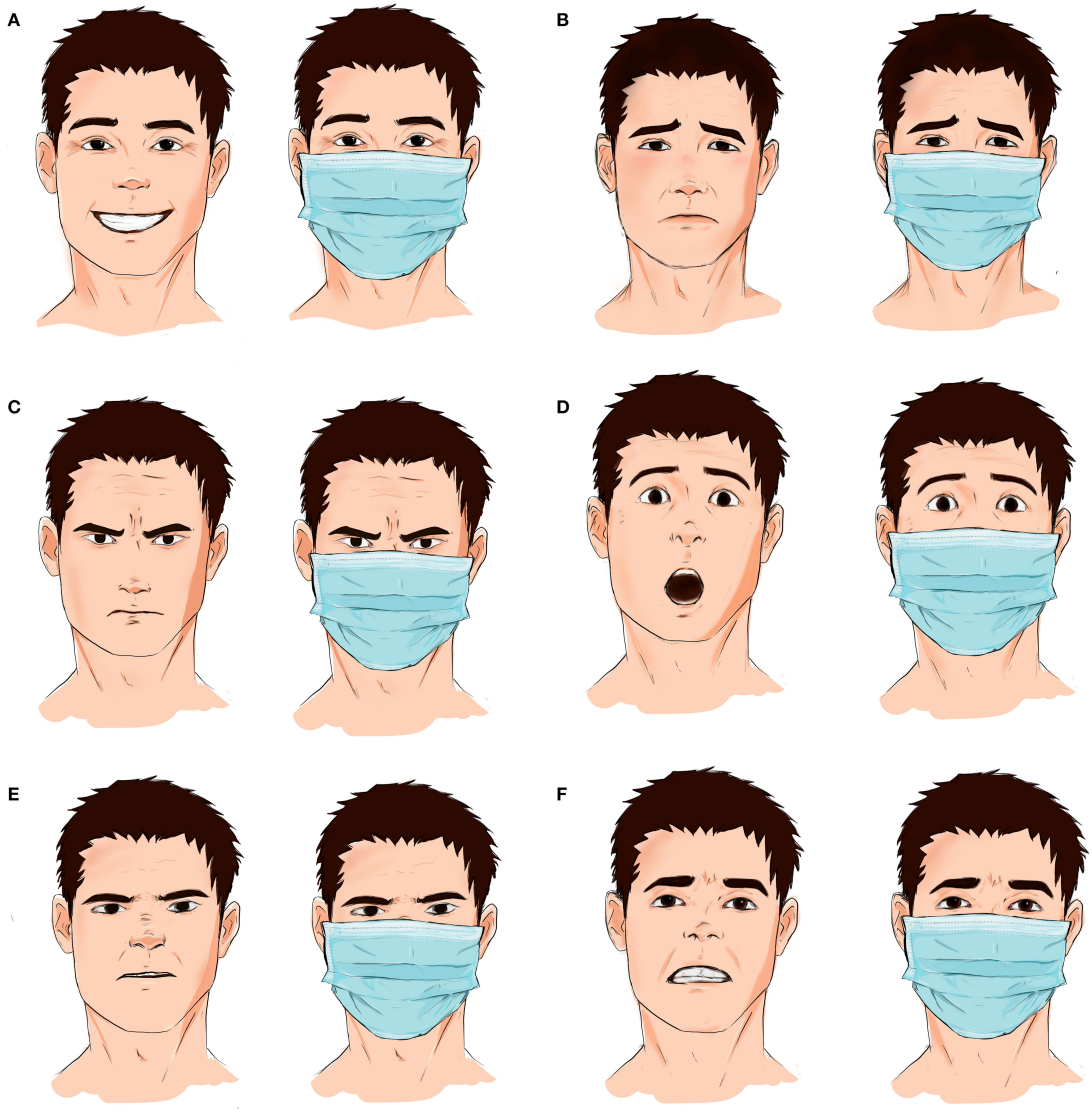
Face masks cover the middle and the lower portions of the face. As such, facial expressions involving the mouth, lips, teeth, and nose are masked during interpersonal communication. **(A)** Happiness is usually perceived when the corners of the lips rise upward. With face masks, happiness can be caught on the face by focusing on the wrinkles at the edge of the eyes. **(B)** Sadness involves movement of the eyebrows, the nasolabial folds, and the corners of the lips; however, the last two are masked by face masks. **(C)** Facial expression of anger emphasizes the downward and central movement of eyebrows, the glaring eyes, and narrowing of the corners of the lips, with the latter getting covered by face masks. **(D)** Expressions of surprise and shock are usually formed of elevated eyebrows and a raised upper lip; only the latter is covered by protective masks. **(E)** Nose wrinkling and raising of the upper lip convey feelings of disgust; however, face masks cover both expressions. **(F)** Feelings of guilt are usually portrayed by slightly upping eyebrows together and stretching the mouth, with the latter getting covered with a face mask.

Despite its crucial protective role, the face mask poses challenges on daily face-to-face communications. Interpersonal communication describes the interaction between two individuals or more through oral or physical (gestures) interactions. Proper application of the protective mask involves covering the mouth and the nose, which muffles sound and makes it challenging to understand speech and some higher-pitched voices. Furthermore, face masks eliminate the roles of the middle and lower face in emotional expression, rendering its action units invisible to the receiving individual (Figure 1). For example, in the physician-patient setting, positive facial expressions play an important role in decreasing the patient's anxiety (10). Therefore, the physician-patient relationship is affected by wearing face masks. Covering the face will reduce the ability of determining the patient's feelings and emotions and affect the physician's measured response to the situation (10). Likewise, the physician's expression of empathy can be missed by the patient. Furthermore, people with special needs and hearing disabilities rely on sign language to communicate. Covering the lower part of the face (nose, cheeks, mouth, tooth, nose, and chin) will adversely affect their understanding of communicated information and make them feel more disabled and ostracized. As a result, emotional perception decreases and the role of the upper face in emotional expression increases in significance.

Nonverbal communication, such as facial gestures and expressions, constitutes 55% of our overall communication (11). The eyes and the mouth are the two main organs that help in reading other's faces. By wearing face masks, people are inclined to focus more on the eyes to be able to understand the facial expressions intended. Eye contact can be used to show empathy and concern for others, to manage feelings, to express interest, or to help with communication. Nevertheless, prolonged eye contact can result in uncomfortable feelings sometimes (11), as it can magnify actual interest in communicated material or convey signs of aggression.

There are a number of populations globally that veil the face for religious or cultural reasons (12). In addition, surgical or cloth face masks have been worn in several East Asian countries since the early 20th century (13). During the 1918 flu pandemic, face masks were commonly worn around the globe (14). After Japan's Great Kanto Earthquake of 1923, firestorms and thick smoke and ash in the air also necessitated face masks. Singapore and Hong Kong suffered flu pandemics in the 1950s and 1960s, and the SARS outbreak of the early 2000s was particularly troublesome for China, Hong Kong, and Taiwan (15). Wearing a face mask became a cultural sign of respect and a social contract toward others. Nevertheless, in the West, the subtraction of nose, mouth, and cheeks during interpersonal communication will necessitate further adaptation.

## Enhancing Communication with Face Masks

**Figure 2:**
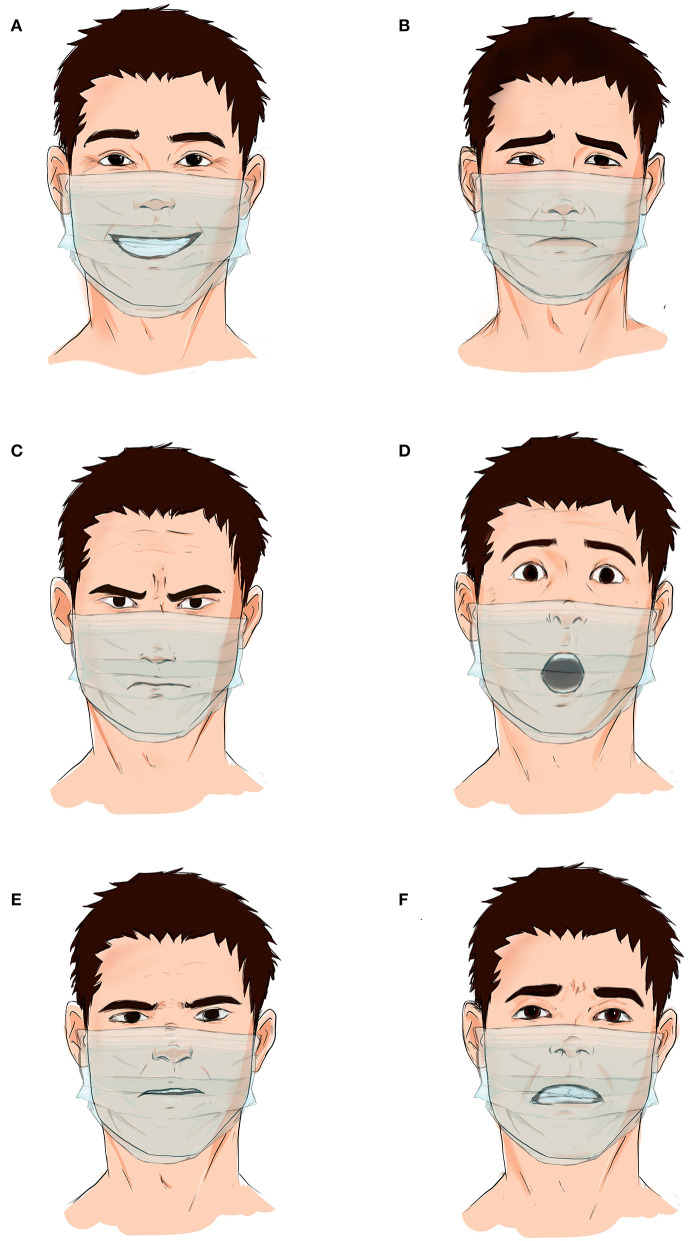
Transparent protective face masks and face shield preserve the importance of facial expressions during interpersonal communication. Feelings of happiness **(A)**, sadness **(B)**, anger **(C)**, surprise **(D)**, disgust **(E)**, and fear **(F)** can easily be noted and picked up through the individual's facial reactions and expressions.

Given the importance of face masks in mitigating the spread of COVID-19, communication adjustments are needed to adapt to the new “normal.” Here, we highlight coping measures that can enhance the quality of interpersonal communication while wearing a face mask:

1. **Raising awareness on the use of face masks and acknowledging the communication challenges that arise as a result in an objective manner**.
  It is important for experts to address the underlying problems and concerns regarding face masks while highlighting their importance as protective equipment against infection (16). This will ease people's acceptance of and commitment to the face mask. Scientists and experts can prevent the spread of false assumptions and empower people by raising awareness on several health challenges and topics through social media, interviews, and podcasts (16).
2. **Utilizing and recognizing the upper face through the eyebrows, eyes, and upper cheeks during interpersonal communication**.
  For example, closing the eyes when agreeing and raising eyebrows when opposing can be adopted in interpersonal settings. The eyebrows, specifically, have received little attention in communication research. Past work has examined the role of eyebrows in emotional expression, nonverbal communication, facial aesthetics, and sexual dimorphism (17–19). For face recognition, the eyebrows may be at least as influential as the eyes. The absence of eyebrows in familiar faces leads to a significant disruption in recognition performance (20). In fact, a significantly greater decrement in face recognition is observed in the absence of eyebrows than in the absence of eyes (20).
3. **Emphasizing the importance of non-verbal communication, such as body language, during communication**.
  For example, people can express their ideas using hand gestures to facilitate the communication process. Non-verbal communications are essential in facilitating the communication process, have a vast influence on the social environment, and can come in different forms, such as facial expressions, body movements, and eye messages, which can support or substitute verbal communication (21).
4. **Paying more attention during interpersonal settings and facing the communication partner directly**.
  This ensures that the communicator has the receiver's attention while nothing is blocking the visual field between them. Synchronous communication is an intended and direct form of communication, which focuses on capturing attention and conveying the needed message. It has been reported that people who communicate through synchronous communication, such as phone or face-to-face communication, perceive the urgency of a situation quicker than those receiving official messages through asynchronous channels, such as text messages (22).
5. **Talking louder and slower in quieter settings**.
  Articulating speech and increasing its volume in a calm setting helps communicators overcome the sound muffling that can result from the face mask. The hierarchy hypothesis asserts that when an individual initially fails to reach social goals through communication, they will continue to try to attain them, but will alter their speech rate and vocal intensity (23).
6. **Relying more on telecommunication for interpersonal interactions**.
  Technological advancements can play a central role in facilitating live connections and interactions between individuals (24). Telecommunication via Skype, Zoom, Facetime, and Cisco Webex was key in keeping the educational, economic, and health sectors alive during the outbreak.
7. **Manufacturing transparent face masks or face shields**.
  People will be able to see each other's facial expressions and emotions without threatening their personal protection (Figure 2). This will also allow people with special needs to communicate easily and understand conversations. The elderly and individuals with hearing impairment rely heavily on facial expressions for communication. Cloth and surgical facemasks hinder their ability to understand and indulge in meaningful conversations (25). The use of transparent face masks will help those individuals read lips and have proper dialogues.
8. **Conducting cross-sectional surveys exploring the effect of face masks on communication**.
  This will help in measuring the impact of the pandemic and wearing face masks on interpersonal communication, quantitatively and qualitatively (26, 27). Research must take into account the cultural differences in communication and the impact of face masks on different societal groups.

## Conclusion

For the time being, face masks are here to stay, as we continue to make efforts to stop the spread of SARS-CoV-2. Nevertheless, identifying the problems and challenges that affect healthy communication while wearing face masks is vital to adapt better to the ensued norm. In addition, developing coping strategies and skills that can ease our communication with face masks is crucial in our efforts to navigate the COVID-19 pandemic and any other pandemic that might erupt in the future.

## Data Availability Statement

The raw data supporting the conclusions of this article will be made available by the authors, without undue reservation.

## Author Contributions

NM and JF conceived the study. NM, MF, and JF wrote the first draft. All authors contributed to the article and approved the submitted version.

## Conflict of Interest

The authors declare that the research was conducted in the absence of any commercial or financial relationships that could be construed as a potential conflict of interest.
